# A mammalian blood odor component serves as an approach-avoidance cue across phylum border - from flies to humans

**DOI:** 10.1038/s41598-017-13361-9

**Published:** 2017-10-20

**Authors:** Artin Arshamian, Matthias Laska, Amy R. Gordon, Matilda Norberg, Christian Lahger, Danja K. Porada, Nadia Jelvez Serra, Emilia Johansson, Martin Schaefer, Mats Amundin, Harald Melin, Andreas Olsson, Mats J. Olsson, Marcus Stensmyr, Johan N. Lundström

**Affiliations:** 1 Department of Clinical Neuroscience, Karolinska Institutet, 17177 Stockholm, Sweden; 2 Donders Institute for Brain, Cognition, and Behavior and Center for Language Studies, Radboud University, 6500 HD Nijmegen, The Netherlands; 3 Department of Psychology, Stockholm University, 10691 Stockholm, Sweden; 4 Department of Physics, Chemistry and Biology, Linköping University, 58183 Linköping, Sweden; 5 Monell Chemical Senses Center, Philadelphia, PA 19104 USA; 6 Department of Biology, Lund University, 22362 Lund, Sweden; 7 Kolmården Wildlife Park, Kolmården, 618 92 Kolmården, Sweden; 8 Department of Psychology, University of Pennsylvania, PA 19104 Philadelphia, USA

## Abstract

Chemosignals are used by predators to localize prey and by prey to avoid predators. These cues vary between species, but the odor of blood seems to be an exception and suggests the presence of an evolutionarily conserved chemosensory cue within the blood odor mixture. A blood odor component, E2D, has been shown to trigger approach responses identical to those triggered by the full blood odor in mammalian carnivores and as such, is a key candidate as a food/alarm cue in blood. Using a multidisciplinary approach, we demonstrate that E2D holds the dual function of affecting both approach and avoidance behavior in a predator-prey predicted manner. E2D evokes approach responses in two taxonomically distant blood-seeking predators, Stable fly and Wolf, while evoking avoidance responses in the prey species Mouse. We extend this by demonstrating that this chemical cue is preserved in humans as well; E2D induces postural avoidance, increases physiological arousal, and enhances visual perception of affective stimuli. This is the first demonstration of a single chemical cue with the dual function of guiding both approach and avoidance in a predator-prey predicted manner across taxonomically distant species, as well as the first known chemosignal that affects both human and non-human animals alike.

## Introduction

In mammals, endogenous odors, such as body odors, urine, feces, or blood, serve as attractants to predators and warning signals to prey species^1^. These types of odors typically transfer information between predator-prey species pairs sharing a long evolutionary history^1^. However, this information flow is normally confined to discrete pairs of predator-prey species but the odor from blood seems to be a more universal cue. Mammalian predators use the odor of blood to home-in on wounded prey^1^, whereas prey species display avoidance behavior and increased vigilance towards conspecific, as well as heterospecific, blood odors^2–7^. This suggests the presence of an old and evolutionarily conserved chemosensory cue within blood odor.

**Figure 1:**
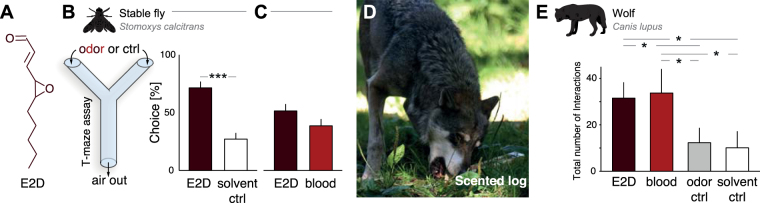
The odor of E2D is sufficient to elicit approach response in blood-seeking animals. (**A**) Chemical structure of *trans*-4,5-epoxy-(**E**)-2-decenal (E2D). (**B**) Schematic drawing of the Y maze olfactory assay used for the fly behavioral experiment. (**C**) Mean percentage of flies choosing between E2D in a background of host odor and host odor only (left) and between E2D and cattle blood both in a host background. (**D**) A wolf displaying biting on the scented log, one of the eleven behaviors present in the ethogram. (**E**) Mean total number of interactions during a session with the four odor stimuli for the wolf pack. Error bars indicate standard error of the mean (SEM). *p < 0.05, ***p < 0.001.

However, the odor of blood is a complex mixture of volatiles, and mammals very seldom respond to a single volatile compound as they do to the naturally occurring odor mixture the volatile belongs to^8–14^. Nonetheless, we recently demonstrated that the volatile chemical *trans*-4, 5-epoxy-(E)-2-decenal (see E2D; Fig. 1a), that is present in mammalian blood is as strong an attractant to four mammalian predator species as the full blood mixture^9^. E2D is generated by lipid peroxidation^15^. This physiological process is present in all mammals and it is very likely that the corresponding volatile metabolites, including E2D, are ubiquitously found in the blood of all mammals. Thus, E2D could indicate the presence of wounded prey to a predator and initiate a behavioral response as efficiently as a natural, complex blood odor.

Olfactory sensitivity to a specific odorant depends on its behavioral relevance for the survival and reproduction rate of individuals of a given species^16,17^. In line with this argument, we recently demonstrated that CD-1 mice (an outbred strain) display extremely high olfactory sensitivity towards this chemical^18^. This indicates that for mice, E2D—like blood—could be an avoidance cue. These observations suggest that high sensitivity to E2D may be evolutionarily conserved across animals and, at least from a behavioral point of view, constitute a key compound of the blood odor cue involved in approach/avoidance responses. This would be rare as, to the best of our knowledge, there is no known chemical cue with the dual function of affecting both approach and avoidance behavior in predator and prey in a predicted manner.

In addition to the two non-human animal studies described above, another study has determined the olfactory detection threshold for E2D in humans. Intriguingly—and as with mice—humans display an extraordinarily low detection threshold of 0.078–0.33 ppt (parts per trillion) for this odorant^15^. Although humans are thought to be opportunistic predators^19^, paleontological data indicate that early primates were small-bodied insectivores^20^ and that predator pressure is likely to be ancient in our clade^19,21^. Moreover, anthropological evidence suggests that more complex forms of large prey hunting in primates increased with the emergence of the genus *Homo* ^19^. Thus, if the chemical cue of E2D is conserved in humans, it is likely in the form of a contextual alarm cue. If true, this would, to our knowledge, constitute the first demonstration of a chemosensory cue that affects behavior in both human and non-human animals alike. However, if such a chemosensory cue does exist, it should be conserved in predator and prey across species, within as well as between phyla.

To address these notions, we used a multidisciplinary protocol that enabled us to assess behavioral responses to E2D across taxonomically distant species. We first determined whether E2D is a key compound of the blood odor cue across taxa by comparing it to the full blood odor in three non-human species of animals. We tested one blood-seeking invertebrate (Stable fly) and one mammalian (Wolf) predator, as well as one mammalian prey species (Mouse). We predicted that both predators would be attracted to E2D to the same degree as to the full blood odor. In addition, we predicted that E2D would serve as an alarm cue in mice by inducing similar avoidance responses as full blood odor. Finally, we addressed whether niche-dependent responses towards E2D are conserved in humans as well. We predicted that as in mice, this odorant would induce a behavioral avoidance response in humans. Moreover, since previous studies have demonstrated that behaviorally relevant odors can affect physiological responses in humans^22–25^, we predicted that E2D would modulate a range of responses associated with the perception of danger.

## Results

First, to determine how an invertebrate blood-seeking animal responds to the odor of E2D, we examined blood chemotaxis of the stable fly, *Stomoxys calcitrans*, a livestock pest of which both sexes require blood meals for survival and reproduction^26^. We assayed flies (n = 35) in a Y-maze setup (Fig. 1B) in which the flies were first provided with a choice between E2D and a near-odorless organic solvent (diethyl phthalate) within the context of a host odor (horse or cattle). Given this choice, the flies significantly preferred E2D (*χ* ^2^(1) = 25.7195, p < 0.001; see Fig. 1C, left panel). Next, we contrasted E2D against real blood (cattle), again within the context of a host odor, and the flies now displayed the same level of approach response to E2D as to the odor of blood, i.e., equating the two odor sources (*χ* ^2^(1) = 1.847, p = 0.17; see Fig. 1C, right panel).

We then sought to determine whether E2D would be sufficient to elicit approach responses typical to those elicited by real blood in a large mammalian predator. To this end, we tested the Eurasian wolf (*Canis lupus*), a gregarious carnivore that relies heavily on olfactory information when tracking and hunting prey^27^. We placed odor-impregnated wooden logs into the wolf packs’ (n = 7) outdoor enclosure to allow the animals to freely interact with the logs. Over a twenty-day experimental period, the wolf pack was presented with logs impregnated with either E2D, real blood (horse), or two control conditions – a fruity odor (iso-pentyl acetate) or the near-odorless organic solvent (diethyl phthalate), while individual interactions with the logs were recorded (see Fig. 1D). There were significant differences in the number of interactions between odorized logs (*χ* ^2^(3) = 17.597, p < 0.001). As hypothesized, the wolves displayed a higher number of interactions when presented with E2D compared to the fruity odor (p < 0.05) and the near-odorless solvent (p < 0.05). There was, however, no significant difference between E2D and the full blood odor mixture (p = 0.997). The wolves also displayed a significantly higher number of interactions when presented with horse blood compared to fruity odor (p < 0.05) and the near-odorless solvent (p < 0.05). The number of interactions did not differ significantly between the fruity odor and the solvent (p = 1.0; see Fig. 1E).

These findings suggest that for blood-seeking invertebrates and carnivorous mammals alike, E2D serves as a conserved chemical cue indicating the presence of blood. In short, we conclude that for both vertebrate and invertebrate blood-seeking animals, the monomolecular odor E2D is sufficient as a chemical cue to initiate an approach behavior that is indistinguishable from that elicited by the complex odor mixture of natural mammalian blood.

**Figure 2:**
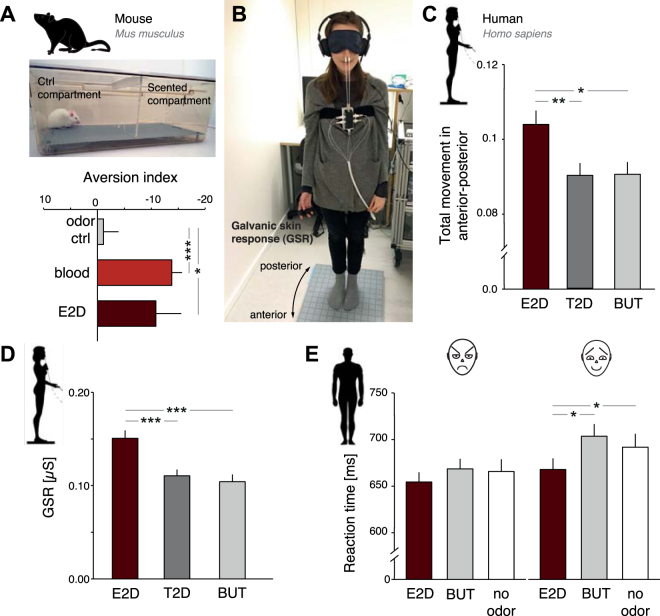
E2D induces avoidance behavior in the prey species mouse and in human participants. (**A**) Upper panel: A mouse displaying aversion towards E2D. Lower panel: mean aversion index of the time spent in the non-odorized compartments. (**B**) A human participant standing on the force platform measuring approach (anterior)-avoidance (posterior). (**C**) Mean total movement (cm) in the anterior-posterior direction as a function of E2D, and the control odors *trans*-2-decenal (T2D) and butanol (BUT). (**D**) Mean GSR signal as a function of E2D, T2D and BUT. (**E**) Mean visual reaction time to affective faces in human participants as function of E2D, BUT, and odorless control. Error bars indicate s.e.m. *p < 0.05, **p < 0.01, ***p < 0.001.

Having concluded that predators and blood-feeding animals use E2D as a chemical cue to locate and approach blood, we next asked whether E2D would induce avoidance responses in prey species. First, we tested the behavioral response of mice (*Mus musculus*), a known prey species for which the odor of blood might indicate the presence of injured conspecifics. We tested predator-naïve CD-1 mice, an outbred strain which has a behavioral phenotype more similar to wild mice compared to inbred strains^28^. We measured time spent on either side of a 2-compartment test arena, with the near-odorless organic solvent (diethyl phthalate) on one side and either E2D, real blood, or a fruity odor (*n*-pentyl acetate) on the other (see Fig. 2A). Both the odor of E2D and the odor of the full blood mixture generated aversive behavior in mice. The mice (*n* = 60) spent significantly more time in the near-odorless compartment compared to both E2D (*V* = 2792.5, p < 0.05) and blood (*V* = 58893.5, p < 0.0001), but not compared to the fruity odor (V = 31595, p = 0.65). As with flies and wolves, the response to E2D and blood did not significantly differ from each other (*χ* ^2^(1) = 2.3726, *p* = 0.12).

Taken together, these experiments confirmed that the mammalian blood odor component E2D elicits approach-avoidance responses in mammalian predator and prey species as well as in blood-feeding invertebrates, in accordance with their ecological niches. Our findings also show that E2D initiates a behavioral response as efficiently as a natural, complex blood odor.

Next, we sought to determine whether E2D is conserved as a chemical alarm cue in humans (*Homo sapiens*) as well. We hypothesized that E2D would serve as a contextual threat and alarm cue that warns the individual of impending danger. According to the well-validated threat-imminence and defense-cascade model, behavioral responses to stimuli that warn the individual of a potential threat in the vicinity are manifested by active avoidance responses, increased arousal, vigilance, and attention^29,30^. We first assessed whether participants would demonstrate an automatic avoidance response towards the E2D odor using a strain gauge force plate (see Fig. 2B). This is a method for measuring approach-avoidance responses in humans by the use of balance-dependent sway in the anterior–posterior (AP) direction^31^. Moreover, we measured physical arousal by means of galvanic skin responses (GSR). Specifically, we hypothesized that participants would demonstrate an avoidance response by initiating a backward leaning motion and elevated physical arousal response towards the E2D odor. To control for odor specificity of the behavioral and autonomic response, we used two control odors, *n*-butanol, frequently used in human olfactory research, and *trans*-2-decenal, a molecule that is structurally similar to E2D, differing only by the presence vs. absence of a functional epoxy group. We matched all three odors on intensity, using weak but detectable iso-intense concentrations. Notably, there were no significant differences between odors in either pleasantness or familiarity. All three odors were rated as neutral in valence (iso-pleasant) and low in familiarity (see Materials and Methods section for ratings and analysis). Participants (*n* = 40) were standing on the force plate blindfolded and E2D, *n-*butanol, and *trans*-2-decenal were birhinally presented using a computer-controlled olfactometer^32^ (see Fig. 2B). Importantly, participants could not anticipate the stimuli onsets, and no mention of blood or blood odor was given either before, during, or after the experiment. Post-experiment control questions showed that no participant had any association with the odor of blood (see Materials and Methods section for more information). In response to 2 s long odor puffs, we observed that E2D increased movement in the AP axis significantly more than both odorant controls, n-butanol (estimate = −0.010851, t(2242) = 2.395, p < 0.05), and trans-2-decenal (estimate = −0.013613, t(2242) = 3.158, p < 0.01), whereas the latter two did not significantly differ from each other in their total AP axis movement (estimate = −0.002762, t(2242) = 0.610, p = 0.81; see Fig. 2C). Subsequent post-hoc tests against zero (no movement) demonstrated that E2D increased movement towards the posterior, a leaning back motion (p < 0.05) whereas both control odors did not differ from baseline (*n*-butanol: p = 0.15, and *trans*-2-decenal(p = 0.31). Similarly, E2D increased the participants’ physical arousal by inducing a significantly higher GSR signal than both control odors (*n*-butanol, estimate = 4.2615, t(2242) = 4.515, p < 0.001, and trans-2-decenal (estimate = −4.0195, *t*_(2242)_ = 4.478, p < 0.001), which in turn did not significantly differ from each other (estimate = 0.2421, t(2242) = 0.256, p = 0.96). Both of these responses, the leaning back motion and the increased physical arousal, are viewed as hallmarks of avoidance behavior in humans^31,33^.

Having established that E2D induced an avoidance response in humans, we next asked if this alarm cue has broader cognitive implications. We tested this by studying if E2D modifies the emotional saliency of visual stimuli by increasing vigilance and attention. Earlier studies have shown that ecologically relevant chemosensory cues, such as body odors, can modify behavioral responses to ambiguous signals of visual threat by making them more similar to unambiguous warning signals^34^. Accordingly, we used a well-validated visual search task^35^ that contained affective facial stimuli while measuring reaction time (RT) and GSR, as the autonomic measure of arousal. We used schematic rather than real faces for three principal reasons: (i) their stereotypical expressions of affective valence, (ii) to limit the influence of previous associations and gender/racial biases that real faces would evoke, and (iii) ensuring low perceptual expertise with the images across participants^32^. These parameters mean schematic faces produce lower variability in evoked arousal than real faces do^32^. In three separate blocks, participants (*n* = 33) were instructed to, as quickly as possible, determine whether all faces in a 3 × 3 array of schematic faces were identical or if one was different while being exposed to either E2D, *n*-butanol, or clean air (same concentrations as in the previous experiment). An angry or a happy face was present as a target among neutral distractor faces; previous studies have demonstrated an angry/threat superiority within this task^32^. As hypothesized, there was a main effect of chemosensory cue on both RT (*F*(2, 64) = 6.168, *p* < 0.01 (see Fig. 2E), and GSR (*F*(2, 64) = 3.241, *p* < 0.05; see Fig. 2D). Pairwise comparisons revealed lower RTs for E2D than for both the odorant control *n*-butanol (p < 0.01), and the odorless control, *p* < 0.05, but the latter two did not significantly differ from each other (p = 0.32). Exposure to E2D, but not *n*-butanol or clean air, speeded RTs to happy faces bringing them to the same level of response time as to that of angry faces. Replicating our previous GSR finding, E2D increased GSR significantly more than *n*-butanol (p < 0.05), and odorless control (p < 0.05), whereas the latter two did not significantly differ (p = 0.625).

## Discussion

Using a multidisciplinary approach, we demonstrate that across taxonomically distant species, for which adaptive behavior towards the odor of mammalian blood is important, one single odorant can elicit approach and avoidance in predator-prey predicted manner. Specifically, our results from non-human animals demonstrate that E2D serves as a signature blood odorant across phylum borders by indicating food in blood-feeding invertebrates, as well as in mammalian predators, while simultaneously cueing danger in mammalian prey to the same extent as the full blood odor. This is unique for two reasons: firstly, mammals do not usually respond to a single volatile compound as they do to the naturally occurring odor mixture the odorant belongs to and, secondly, to the best of our knowledge, there is no known single odorant with the dual function of affecting both approach and avoidance in a predator-prey predicted manner. From an evolutionary point of view, behavioral adaptation towards invariant chemical cues present in blood may have constituted a cost-effective way for predators to locate wounded prey from different species, and for preys to avoid novel and potentially dangerous environments containing conspecific or heterospecific blood. It is possible that the selection pressure for this chemical cue is old, likely predating several of the other known species-specific chemical cues that have been shaped by predator-prey interactions.

We further demonstrate that E2D affected several responses in humans despite being perceived as an odor stimulus of low intensity, and neutral valence. First, E2D triggered a postural avoidance, which was in line with our hypothesis that humans, like mice, would display a prey-typical response to this odorant. The increase in the participants’ physical arousal in two independent experiments further corroborated this observation. Finally, the enhanced efficiency in detecting emotional visual stimuli demonstrated that the effect of E2D went beyond simple peripheral responses by modulating more complex cognitive functions prototypical for a defensive system. This suggests that E2D, at least in humans, functions as a contextual alarm cue affecting afferent systems, which in turn leads to increased stimulus receptivity and visual processing.

Due to ethical restrictions concerning pathogen transfer, direct comparison between E2D and blood was not possible in human participants. However, we believe that our comparative approach justifies and makes it likely that this chemical cue is conserved in humans and non-human animals alike. Predator-prey interactions have been a major force in shaping olfactory perception in the animal kingdom^1^, and are likely important in the hominin lineage^19^. Cross-species signals are well-established in other modalities^19^ and olfaction is no exception^17^. For example, both elephants and a large number of moths share parts of their sex pheromones, and many alarm pheromones and other chemical cues are shared in species within the same prey guild^17^. However, unlike these types of chemical stimuli, the odor of blood is characterized by a rare universality^1–7^. We propose that the driving force behind this is the ecological relevance of blood odor for survival, and that E2D may be an invariant cue shared across species. Invariant cues across animals are well-established in both auditory and visual perception^19,33^. For example, many species, including mammals, birds, reptiles, and human adults as well as infants, display strong gaze sensitivity^19,34^. Similar to the widespread use of gaze information, a liquid such as blood, omnipresent in all mammals, would be an ideal candidate for the adaptation towards invariant food and alarm cues shaped by the long evolution of mammalian predator-prey interactions. However, based on our results we cannot draw valid conclusions whether our data support the notion of an evolutionarily preserved common chemical cue or the notion of convergent evolution leading to comparable behavioral responses in different taxa. Moreover, it is important to point out that we cannot completely exclude the possibility that these responses may be, at some stage of development, modulated or initiated by learning processes. Future studies need to elucidate whether nature or nurture is creating these behavioral responses.

In our study, concentrations above detection threshold were used in all experiments to elicit clear behavioral responses. Thus, future studies need to establish the lowest concentration of E2D needed to trigger a behavioral response. Furthermore, whether these across-species behavioral responses are mediated by a shared mechanism needs to be determined by studies targeting the receptor(s) and the underlying neural process. Moreover, although numerically lower, E2D did not significantly decrease RT for the angry face stimuli. This could be due to low power or a lack of effectiveness of E2D to make the angry face more negative. However, we believe the more likely explanation is that this is the result of a floor effect caused by the anger superiority effect. Also, future studies should assess a wider range of emotional facial stimuli to better evaluate the specificity of the E2D effect. It is important to note that our results do not exclude that other blood-related odorants can elicit similar responses as demonstrated above. The full blood odor is a complex mixture with several possible candidates and future studies are needed to determine their potential effects. However, few examples exist of monomolecular odors eliciting behavioral responses similar in magnitude as those observed for the full natural mixture. Indeed, even the widely used fox odor component trimethylthiazoline (TMT), which causes fear and avoidance responses in rodents, demonstrates effects that are clearly weaker than those in response to the real fox faeces odor^35^. The majority of field studies demonstrate that monomolecular predator odors convey very limited information, and require additional odorants from the natural mixture to be efficient^36^. Thus the uniqueness of these findings makes the likelihood of finding additional blood odor candidates with the same characteristics less likely.

Taken together, our results strongly suggest that E2D is a blood signature substance that serves as an approach-avoidance cue across phylum borders; it elicits approach responses in blood-feeding invertebrates as well as in mammalian predators, while eliciting avoidance behavior in mammalian prey species. These results demonstrate the existence of a cross-species food- and alarm cue that affects behavior in both human and non-human animals alike.

## Material and Methods

### Participants

In Experiment 1 (Fly), a total of 35 Stable flies (*Stomoxys calcitrans*) were used. In Experiment 2 (Wolf), seven uncastrated adult male Wolves (*Canis lupus*) forming a wolfpack maintained at Kolmården Wildlife Park were studied within their normal outdoor enclosure out of sight of visitors. In Experiment 3 (Mouse), a total of 60 individually housed CD-1 mice (*Mus musculus*) were studied. All mice were between 90–130 days of age at study onset and laboratory-born without any exposure to natural predators. In Experiments 4 and 5 (Human), healthy adult participants (*Homo sapiens*) without sensory deficit participated. In Experiment 4, a total of 40 participants participated (mean age 24; of which 20 women) and in Experiment 5, a total of 37 participants participated but data from 4 individuals were excluded due to technical problems during recording of the psychophysical data. This resulted in a final sample of 33 participants (mean age 26; of which 20 women).

### Odor Stimuli and presentation

All synthetic chemicals were obtained from commercial sources (Sigma, www.sigmaaldrich.com, and aromaLAB, Freising, Germany) and were of the highest purity available and all odors were diluted in near-odorless diethyl phthalate (CAS# 84-66-2). Also, diethyl phthalate was used as the near-odorless organic control solvent in Experiment 1, 2, and 3. Importantly, this odorant is neither aversive nor attractive for either the current or previously tested species^9^. In Experiment 1, 4, and 5, *trans*-4, 5-epoxy-(E)-2-decenal (E2D; CAS #134454-31-2) was diluted down to a 10% v/v concentration from a 5 mg/ml stock solution and in Experiment 2 and 3, E2D was diluted down to 1% concentration from stock solution. In Experiment 1, 2, and 3, undiluted animal blood (horse, mouse, cat, and human) was used. Moreover, different monomolecular odors were used as controls in the various experiments. In Experiment 2, iso-pentyl acetate (CAS# 123-92-2) was diluted down to 0.1% concentration from neat content; in Experiment 3, *n*-pentyl acetate (CAS# 628-63-7) was diluted down to 0.0001% concentration; in Experiment 4 and 5 (Human), *n*-butanol (CAS# 71-36-3) was diluted down to a 0.16% v/v concentration; in Experiment 4, *trans*-2-decenal (CAS# 3913-81-3) was diluted down to 0.125% v/v. Odor concentrations were selected to be above detection limits for each species but at a weak perceptual concentration. For Experiments 4 and 5, these concentrations were selected from a range of concentrations after a pilot experiment (n = 10 particpants) indicated that these were of weak, yet clearly discernable iso-intense and iso-pleasant concentrations. Participants were asked to rate the intensity and familiarity of the odors on a visual analog-scale from very low (0) to very high (10), and their pleasantness on a scale from very unpleasant (-5) to very pleasant (5), with the middle (0) indicating neutral valence. Repeated measure ANOVAs of the perceptual ratings in Experiments 4 showed that there were no significant differences in intensity between E2D (M = 4.05, SD = 1.96), trans-2-decenal (M = 3.83, SD = 1.92) and n-butanol (M = 3.68, SD = 1.90) (F(2,78) = 0.52, p = 0.60). The same was true for ratings of familiarity (E2D: (M = 3.63, SD = 2.49; trans-2-decenal: M = 3.50, SD = 2.71; n-butanol (M = 3.83, SD = 2.95; F(2,78) = 0.277, p = 0.76); and pleasantness (E2D: M = 0.33, SD = 2.1; n-butanol: M = −0.15, SD = 2.02; trans-2-decenal: M = −0.28, SD = 1.57; F(2,78) = 1.44, p = 0.24). Likewise, paired-sample t-tests between E2D and n-butanol in experiment 5 did not show any significant difference in intensity (E2D: M = 3.87, SD = 2.09; n-butanol: M = 3.98, SD = 2.09; t(32) = 0.25, p = 0.81) or pleasantness (E2D: M = 0.36, SD = 1.71; n-butanol: M = −0.03, SD = 1.68; t(32) = 1.01, p = 0.32). For flies, stimulus delivery followed established protocols^37^. For wolves, odors were presented on wooden logs as previously described with the exception that they were secured to a chain to limit the range of allowed movement^9^ and for mice, each compartment had a petri dish with an impregnated filter paper with the odor in question located centrally underneath the perforated cage floor. For humans, all odors were presented birhinally using a computer-controlled olfactometer with a known rise-time of 160 ms^38^ and a total flow-rate of 1.5 liter/minute (l/min) per channel for Experiment 4 (3 l/m for Experiment 5) and inserted into an ongoing 0.3 l/min constant flow to avoid any tactile sensation of the odor onset. This means that total airflow per nostril was never higher than 1.75 l/min, a flow significantly lower than airflows known to elicit nasal irritation^38^, and flow was intermittently terminated using a jittered design between trials to allow the mucosa to rehydrate. In Experiment 4, to avoid sniff-dependent effect on balance measures, odor delivery was synchronized to the participants’ inhalation by means of sniff-triggering of the olfactometer in that the olfactometer was triggered at the nadir of the participants’ exhalation, determined by a nasal cannula connected to a piezoelectric pressure sensor, i.e. the participant could not anticipate the odor onset. In Experiment 5, odors were delivered on average 1.5 s before (jitter 0.5–2 s) the onset of the visual task to prime the visual response.

### Psychophysiological measures

Posturography measures used to derive time series of the center of pressure for the anterior-posterior (AP) direction were obtained by means of an AccuSway force platform (AMTI, Watertown, MA). The collected data were calibrated on an individual level and filtered with a low-pass filter using a cut-off frequency of 5 Hz. Average AP-sway for each condition was formed by subtracting movement 0.5 s pre-stimulus from the average 1 s after each trial to capture event-related responses. GSR was obtained and analyzed similar to previous studies^39^ with the exception being that the average electrodermal activity 0.5 s pre-stimulus was subtracted from the peak response within 8 s post-stimulus onset to reflect stimulus-evoked responses. Heart rate was also measured in experiment 5 but incorrectly acquired due to technical problems.

### Behavioral measures

For flies, preference tests between natural blood and E2D with host odor as context, as well as E2D and natural blood versus diluent only within the host (cattle and horse) context, were performed in a standard Y-maze setup^38^ where number of flies selecting each odor was recorded. For wolves, scented logs were placed within the enclosure for 3 h in the morning and 3 h in the afternoon with a total of 20 repetitions with intertrial intervals of at least one day. Number of interactions was recorded according to a previously used ethogram^9^ with one addition measure noted, guarding. Mice were tested in a two-compartment test arena and time spent within each compartment, out of a total of 600 seconds, was recorded^40^. For humans, sensory functions were established using a visual Snellen test in Experiment 5 and a 5 items 4-alternative cued odor quality identification test (all participants, ≥4 correct ID responses) in both Experiment 4 and 5. Exclusion criteria were self-reported health problems demanding medication as well as psychiatric, neurological, and other diagnoses affecting emotional regulation or the vestibular system, as well as being a habitual smoker. In Experiment 4 and 5, subjective ratings of intensity and pleasantness of all the odors were averaged from 3 repeated ratings obtained during each experiment. In Experiment 5, participants were presented with 3 × 3 matrices of schematic faces demonstrating prototypical emotional features of neutral, angry, or happy faces^32^; half were entirely composed of neutral faces (lures) and the remaining matrices were divided between matrices where one face at a random position consisted of either an angry face or a happy face (target). Participants’ task in each trial was to determine, as fast as possible, whether the matrix presented to them consisted of all the same faces or if one face was different. Each trials was initiated by a visual fixation cross in the center of the screen which stayed on for an average of 2 s (jitter: 1.5–3 s) and was immediately replaced by a matrix that remained until participant response or for a maximum of 3 s.

### Statistical Analysis

For Experiment 1 (Fly), the contrasts between E2D and odorless solvent and between E2D and blood were tested using χ^2^ tests with Yates’ correction for continuity. For Experiment 2 (Wolf), the total number of interactions per stimulus was tested using Friedman tests with Nemenyi post-hoc test. In Experiment 3 (Mouse), Wilcoxon signed-rank test was used for measuring time spent in the odorized compartments compared to the non-odorized compartment. The comparison between odorized compartments was tested using χ^2^ tests with Yates’ correction for continuity. Statistical differences in GSR and movement in the AP axis between conditions in Experiment 4 (Human), were assessed using separate mixed-linear ANOVA models with the subject factor as a random effect and GSR values and AP movement as a fixed effect. For movement, subsequent post-hoc tests against zero (no movement), using Holm-Bonferroni correction, were performed for each condition to assess whether there was a significant movement forward (positive values) or backwards (negative values) in response to each odor. For GSR, subsequent corrected pair-wise post-hoc tests were performed to assess response-differences between conditions. For Experiment 5 (Human), RT and GSR were assessed using repeated measure ANOVAs with within-subject factors Facial expression (angry, happy) and Odor (E2D, *n*-butanol, odorless air). Main effects were followed by pair-wise post-hoc tests. The alpha level was set to 0.05 in all tests and all Student’s *t*-tests and ANOVAs were two-tailed, the analyses were performed in R, and the mixed-effects models used the *lme4* package.

### Ethical and data availability Statement

Informed written consent was obtained from all human participants and all study procedures and protocols were, in respect of the vertebrate animals, approved by the respective regional ethical review board (Wolf: Jordbruksverket, Dnr. 5.2.19-5974/15; Mice: Linköpings djurförsöksetiska nämnd, Dnr. 76/12; Human: Stockholm’s Ethical Review Board, Dnr. 2016/1692-31/4) and all study procedures and protocols were carried out in accordance with all relevant guidelines and regulations. Individual depicted in Fig. 2B provided consent for inclusion of image in publication. All data directly associated with this publication will upon request be shared without restrictions.
